# Pre‐treatment with D942, a furancarboxylic acid derivative, increases desiccation tolerance in an anhydrobiotic tardigrade *Hypsibius exemplaris*

**DOI:** 10.1002/2211-5463.12926

**Published:** 2020-07-22

**Authors:** Koyuki Kondo, Masaru Mori, Masaru Tomita, Kazuharu Arakawa

**Affiliations:** ^1^ Institute for Advanced Biosciences Keio University Tsuruoka, Yamagata Japan; ^2^ Systems Biology Program Graduate School of Media and Governance Keio University Fujisawa Japan; ^3^ Exploratory Research Center on Life and Living Systems National Institutes of Natural Sciences Okazaki Japan

**Keywords:** anhydrobiosis, desiccation tolerance, oxidative stress, proteomics, tardigrade

## Abstract

The tardigrade *Hypsibius exemplaris* can undergo anhydrobiosis. Several chemicals that inhibit successful anhydrobiosis in *H. exemplaris* have been identified, and these chemicals inhibit the activity of signaling molecules. In the present study, we investigated whether upregulation of the activity of these signaling molecules could improve desiccation tolerance of *H. exemplaris*. Pre‐treatment with an indirect activator of AMP‐activated protein kinase [AMPK; which directly inhibits mammalian NAD(P)H dehydrogenase [quinone] 1 [NQO1] of mitochondrial complex I (D942)] significantly improved desiccation tolerance of *H. exemplaris*, whereas a direct activator of AMPK did not. To elucidate the underlying molecular mechanisms, we examined the proteome of tardigrades treated with D942. Two proteins, putative glutathione *S*‐transferase and pirin‐like protein, were upregulated by treatment. Both of these proteins are known to be associated with the response to oxidative stress. One of the downregulated proteins was serine/threonine‐proteinphosphatase 2A (PP2A) 65‐kDa regulatory subunit A alpha isoform, and it is interesting to note that PP2A activity was previously suggested to be required for successful anhydrobiosis in *H. exemplaris*. Taken together, our results suggest that D942 treatment may partially induce responses common to those of desiccation stress. The identification of a chemical that improves desiccation tolerance of *H. exemplaris* may facilitate further investigation into desiccation tolerance mechanisms.

## Graphical abstract

The tardigrade *Hypsibius exemplaris* has low tolerance to direct exposure to 50% relative humidity (RH) without preconditioning in a high RH environment. However, tardigrades pre‐treated with D942, a furancarboxylic acid derivative, partially acquired desiccation tolerance against 50% or even lower RH without preconditioning. Proteomic analysis of tardigrades treated with D942 indicated that responses to oxidative stress may be one of the underlying molecular mechanisms.

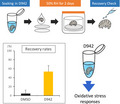

**Abbreviations**
AMPK: AMP‐activated protein kinase
NQO1: NAD(P)H dehydrogenase [quinone] 1
PP1: protein phosphatase 1
PP2A: protein phosphatase 2A
RH: relative humidity

Certain tardigrade species can tolerate extreme desiccation in a state referred to as anhydrobiosis. The desiccation strategy of anhydrobiotic tardigrades can be largely divided into two categories: preconditioning‐requisite and preconditioning‐unrequisite [1]. Tardigrades living in rapidly desiccating environments such as *Ramazzottius varieornatus* constitutively express anhydrobiosis‐related proteins, whereas less tolerant species such as *Hypsibius exemplaris* require exposure to a high relative humidity (RH) environment for a certain period to prepare cellular conditions prior to facing extreme desiccation [2, 3]. *Hypsibius exemplaris* requires *de novo* gene expression for successful anhydrobiosis and shows dramatic changes of transcriptome [2, 3]. In previous studies, we identified several signaling molecules, including protein phosphatase (PP) 1 and PP2A (PP1/PP2A) and AMP‐activated protein kinase (AMPK), required for successful anhydrobiosis in *H. exemplaris*, using chemicals for which the target molecules have been validated in mammals [2, 4]. All identified chemicals so far have been inhibitors and, once tardigrades were pre‐treated with those inhibitors and subsequently exposed to desiccation stress, anhydrobiotic survivals were significantly impaired. We thus hypothesized that pre‐treatment with the activator of the target molecule of the identified chemical inhibitor could oppositely improve desiccation tolerance in *H. exemplaris*. To test this hypothesis, in the present study, we investigated the effects of AMPK activators because we recently showed that AMPK inhibitor (compound C) inhibits anhydrobiotic survivals in *H. exemplaris* [4]. We tested direct and indirect AMPK activators, and showed that only an indirect activator that directly inhibits NAD(P)H dehydrogenase [quinone] 1 (NQO1) of mitochondrial complex I in mammals (referred to as D942) [5] improved desiccation tolerance. In the tardigrades pre‐treated with D942, no significant elevation of kinase activity of AMPK was observed, and there was no apparent presence of *nqo1* genes in the *H. exemplaris* genome, indicating that increased desiccation tolerance is the result of currently unknown mechanisms. Proteomic analysis suggested that responses to oxidative stress may be one of the underlying molecular mechanisms. Although the result did not directly support the initial hypothesis, we identified the chemical that improves desiccation tolerance in *H. exemplaris*. Further investigations aiming to elucidate the mechanisms that improve the desiccation tolerance of this chemical could lead to a deeper understanding of the molecular basis of desiccation tolerance and anhydrobiosis in tardigrades.

## Materials and methods

### Animals

The Z151 strain of *H. exemplaris* was purchased from Sciento (Manchester, UK) and maintained at 18 °C. *Thulinius ruffoi* were originally collected in Poland by Ł. Michalczyk, and maintained in our laboratory at 15 °C. Tardigrades were reared on 2% agar plates overlaid with Volvic water (Danone SA, Paris, France) containing 1% *Chlorella vulgaris* (Chlorella Industry Inc., Tokyo, Japan) as food. Water and food were replaced once a week.

### Desiccation treatment

The procedure was performed as described previously [4]. Briefly, tardigrades were dropped onto a nylon filter (Membrane Solutions, Auburn, WA, USA) placed on a filter paper (Thermo Fisher Scientific, Waltham, MA, USA) in a plastic dish with total of 125 μL water, and then immediately transferred in a sealed plastic box adjusted to 50% or 10% RH and desiccated for 2 days at 18 °C. The desiccated tardigrades were subsequently rehydrated with 2 mL of water and recovery rates at 1 h after rehydration were calculated. Animals showing spontaneous movements or responses to touch stimuli were judged as recovered. Tardigrades desiccated at 10% RH were subjected to 97% RH for 1 day before rehydration with water. Aqueous glycerol solution was used for generating 50% and 97% RH (glycerol; Nacalai Tesque, Kyoto, Japan) [6] and activated silica gel was used for 10% RH. The actual RH was checked using a hygrometer (HN‐CHPR; Chino Corp., Tokyo, Japan).

### Chemical treatment

The procedure was performed as described previously [4]. Briefly, AMPK activator, D942 (D942; Santa Cruz Biotechnology, Santa Cruz, CA, USA) was dissolved in dimethylsulfoxide special grade (FUJIFILM Wako Pure Chemical Corporation, Osaka, Japan) and AICAR (AdipoGen, San Diego, CA, USA) was dissolved in fresh Milli‐Q (MQ) water (Merck, Darmstadt, Germany) as a stock solution (500 and 34.9 mm, respectively) and all were stored at −20 °C. Chemical solutions at the appropriate concentrations were prepared by diluting stock solutions in MQ water just prior to chemical treatment. The final concentration of dimethylsulfoxide in D942 solution was adjusted to 1%. As a control, 1% dimethylsulfoxide solution or fresh MQ water was used. Tardigrades were transferred with 3 μL MQ water into 50 μL of chemical solution of the defined concentration and thus tardigrades were exposed to slightly diluted concentration of the chemicals (94% of the defined concentration). Tardigrades were incubated in the chemical solution for 24 h at 18 °C. Tardigrades in 53 μL of solution were then dropped onto a nylon filter placed on a filter paper, followed by the addition of 72 μL MQ water (total volume: 125 μL) and then subjected to desiccation treatment as described above. Approximately 15 animals in triplicate were used to check the effects of chemicals on the recovery rates after desiccation treatment. For specimens subjected to proteomics, the volumes of chemical solutions and MQ water were modified accordingly on a larger scale as a result of the larger number of specimens.

### AMPK kinase activity assay

AMPK kinase activity was measured using an AMPK Kinase Assay kit (CycLex, Medical & Biological Laboratories Co., Ltd., Nagoya, Japan) in accordance with the manufacturer's instructions. One thousand tardigrades treated with 1 mm of D942 or 1% dimethylsulfoxide for 24 h and 34.9 mm of AICAR or MQ water for 5 h were washed thoroughly, then collected in a 1.5‐mL tube with 5 μL of MQ water and 95 μL of lysis buffer (20 mm Tris‐HCl, pH 7.5; Sigma‐Aldrich, St Louis, MO, USA; protease inhibitor, Complete, EDTA‐free; Roche Life Science, Basel, Switzerland) and stored at −80 °C. Active tardigrades without chemical treatment (untreated) were also collected. Frozen samples were subjected to a freeze–thaw cycle five times and subsequently sonicated at 4 °C for 20 min using a Bioruptor II (BM Equipment Co., Ltd, Tokyo, Japan). Homogenates were centrifuged at 16 000 ***g*** for 10 min and supernatant was collected in new 1.5‐mL tube. The protein concentration was measured using a PIERCE BCA protein assay kit (Thermo Fisher Scientific) and absorbance at 534 nm was measured using a TECAN plate reader (Tecan, Männedorf, Switzerland).

### Similarity search

A similarity search for the amino acid sequence of human *nqo1* against the protein database of *H. exemplaris* (nHd.3.1.5.proteins) was performed using blastp [7].

### Sampling for proteomics

Active tardigrades without chemical treatment (untreated) and tardigrades treated with 1% dimethylsulfoxide or 1 mm of D942 for 24 h were collected in biological triplicates. Each sample contained 500 individuals.

### Protein extraction and quantification

The procedure was performed as described previously [4]. Briefly, protein extraction was performed on ice with pre‐chilled lysis buffer (12 mm sodium deoxycholate; SDC; FUJIFILM Wako Pure Chemical Corporation), 12 mm sodium *N*‐dodecanoylsarcosinate (FUJIFILM Wako Pure Chemical Corporation), protease inhibitor (Complete, EDTA‐free; Roche Life Science), phosphatase inhibitor (PhosSTOP Phosphatase inhibitor cocktail; Roche Life Science) in 100 mm triethylammonium bicarbonate buffer (pH 8.5; Sigma‐Aldrich). Five hundred specimens in a 1.5‐mL tube were carefully ground using a plastic grinder (Biomasher II; Nippi Inc., Tokyo, Japan) with 200 μL of lysis buffer. Homogenates were sonicated at 4 °C for 20 min using a Bioruptor II (BM Equipment Co., Ltd). The protein concentration was measured using a PIERCE BCA protein assay kit (Thermo Fisher Scientific) and absorbance at 534 nm was measured using a TECAN plate reader (Tecan).

### Sample preparation for proteomics

The procedure was performed as described previously [4]. Briefly, whole lysates of *H. exemplaris* were subjected to reduced alkylation with dithiothreitol (FUJIFILM Wako Pure Chemical Corporation) at room temperature for 30 min, followed by another 30 min of incubation in dark with iodoacetamide (FUJIFILM Wako Pure Chemical Corporation). After five‐fold dilution with 100 mm triethylammonium bicarbonate buffer, samples were incubated with lysyl endopeptidase (FUJIFILM Wako Pure Chemical Corporation) for 30 min and subsequently with sequencing grade modified trypsin (Promega, Madison, WI, USA) for 16 h at room temperature to digest whole proteins into peptide fragments. The whole protein digests were treated with C18 StageTip for desaltation, dried under reduced pressure and stored at −30 °C until LC‐MS/MS measurements [8].

### LC‐MS/MS

An UltiMate 3000 nanoLC pump (Dionex Co., Sunnyvale, CA, USA) and a LTQ orbitrap XL ETD (Thermo Electron, Waltham, MA, USA) was used for shotgun proteome analysis of LC‐MS/MS. The dried samples were dissolved in trifluoroacetic acid/acetonitrile/water (0.1 : 5 : 95, v/v/v) and injected into the spray needle column (Reprosil Pur C18‐AQ 3 μm, 100 μm inner diameter, 650 mm length, approximately 5 μm tip inner diameter). Peptide separation in the column was achieved via linear gradient elution using the mixture of two mobile phases (A) acetic acid/dimethylsulfoxide/water (0.05:4:96, v/v/v) and (B) acetic acid/dimethylsulfoxide/ACN (0.05:4:96, v/v/v) at the total flow rate of 500 nL·min^−1^ [(A) + (B) = 100%; (B) 0–0% in 3 min, (B) 0–45% in 240 min, (B) 45–80% in 5 min, (B) 80–80% in 5 min, (B) 0–0% in 25 min]. The separated peptides were sequentially ionized via electrospray mode at 2600 V and injected into an Orbitrap mass spectrometer (Thermo Fisher Scientific) for detection as peptide ions, followed by sequential injection of the top 10 signal peaks into a linear ion trap mass spectrometer to acquire the product ion mass spectrum by collision‐induced dissociation.

### Protein identification

The LC‐MS raw files were analyzed using maxquant, version 1.6.3.4 [9, 10] in conjunction with an in‐house protein sequence database of *H. exemplaris* (Hypsibius_dujardini_nHd.3.1.5.protein) for the identification of peptides or proteins in samples (false discovery rate < 1%) and for peak area integration of mass chromatograms derived from the identified peptides. Each peak area value between different LC‐MS runs was normalized with the peak areas of all identified peptides. The relative expression levels of peptides were calculated by the peak area on each LC‐MS run divided by the mean of the same sequence peptide on all LC‐MS runs, and the expression level of each protein was estimated from the median of the relative expression level of the unique peptide yield from each protein.

### Reduced glutathione (GSH) and oxidised glutathione (GSSG) quantification

GSH‐to‐GSSG ratios were measured using a GSSG/GSH Quantification kit (Dojindo Molecular Technologies, Inc., Kumamoto, Japan) in accordance with the manufacturer's instructions. Two thousands of active tardigrades, *H. exemplaris* without any chemical treatment (untreated) or those treated with 1 mm D942 or 1% dimethylsulfoxide for 24 h were collected in a 1.5‐mL tube with 8.5 µL of MQ water, and stored at −80 °C. After 8.5 µL of 10% 5‐sulfosalicylic acid, 2‐hydrate (Kishida Chemical Co., Ltd, Osaka, Japan) was added to frozen samples, these were subjected to a freeze–thaw cycle five times and subsequently sonicated at 4 °C for 20 min using a Bioruptor II (BM Equipment Co., Ltd.). To dilute 5‐sulfosalicylic acid, 2‐hydrate, 170 µL MQ water was added to homogenates and these were centrifuged at 16 000 ***g*** for 10 min. Then, the supernatant was separately collected in new 1.5‐mL tubes for total GSH and GSSG quantification. Absorbance at 405 nm was measured using a TECAN plate reader (Tecan).

### Statistical analysis

To detect a significant difference in recovery rates after desiccation treatment, Student's *t*‐test or a Tukey–‐Kramer test was performed. A significant difference in AMPK kinase activity was detected by the Tukey–Kramer test. All reported error bars represent the SD. *P* < 0.05 was considered statistically significant. Among all of the proteins, those that were detected as biological triplicates were used for statistical tests. The amount of each protein in D942‐treated or dimethylsulfoxide‐treated groups was compared with that in the untreated group by Student's *t*‐test. Those with a significant change only in the D942‐treated group were regarded as differentially regulated proteins. To improve reliability, we set strict criteria for identifying significant proteins from differentially regulated proteins: (a) more than two‐fold up‐ or downregulation of differentially regulated proteins compared to the untreated group and (b) a change of the amount of protein in the dimethylsulfoxide‐treated group in the range 0.8–1.2 compared to the untreated group.

## Results

### AMPK indirect activator increased desiccation tolerance in *H. exemplaris*

**Fig. 1:**
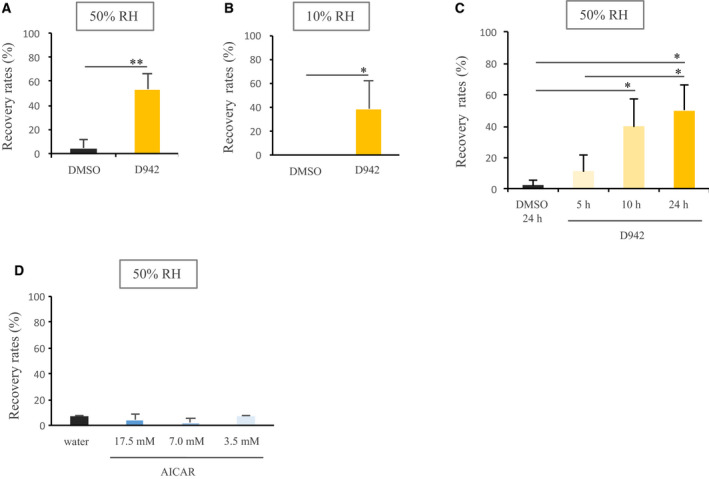
Effects of D942 and AICAR on desiccation tolerance. Recovery rates of tardigrades, *Hypsibius exemplaris* pre‐treated with D942 (1 mm) or 1% dimethylsulfoxide for 24 h and subsequently desiccated at 50% RH (A) or 10% RH (B) for 2 days. (C) Recovery rates of tardigrades pre‐treated with 1% dimethylsulfoxide for 24 h or D942 (1 mm) for 5, 10 or 24 h, then desiccated at 50% RH for 2 days. (D) Recovery rates of tardigrades pre‐treated with AICAR at different concentrations or MQ water for 24 h and subsequently desiccated at 50% RH for 2 days. *Statistical significance between samples (Student's *t*‐test or Tukey–Kramer test; *P* < 0.05). Data represent the mean ± SD; *n* = 3; 15 animals each.

To investigate whether upregulation of AMPK activity improved desiccation tolerance in *H. exemplaris*, we investigated the effects of AMPK indirect activator (commercial name: AMPK activator, D942; direct inhibitor of mammalian NQO1 of mitochondrial complex I; referred to here as D942) on recovery rates after desiccation stress. Compared to dimethylsulfoxide‐treated controls, recovery rates in D942‐treated group were significantly higher after 2 days of desiccation at 50% RH (Fig. 1A). Even when animals were directly exposed to 10% RH and desiccated for 2 days, some of them were recovered in D942 group (Fig. 1B). We next investigated the effects of various soaking periods on recovery rates after 2 days desiccation at 50% RH. Ten and twenty‐four hours of soaking in D942 solution significantly increased recovery rates, whereas 5 h of soaking did not show such positive effects (Fig. 1C). We also investigated another AMPK activator that directly activates AMPK (commercial name: AICAR; AMP analog; referred to here as AICAR). Intriguingly, pre‐treatment with AICAR scarcely improved recovery rates of tardigrades subjected to 50% RH for 2 days, at any concentration (Fig. 1D and Fig. S1). Furthermore, we examined the effects of D942 on desiccation tolerance in desiccation intolerant species *T. ruffoi* and no effects were observed (Fig. S2).

### AMPK kinase activity measurement

**Fig. 2:**
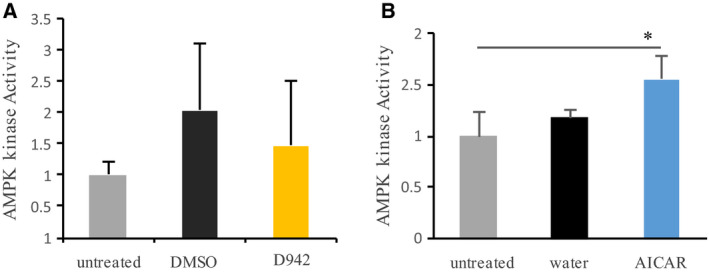
AMPK kinase activity. AMPK kinase activity in homogenates extracted from tardigrades, *Hypsibius exemplaris* treated with D942 (1 mm) for 24 h (A) or AICAR (34.9 mm) for 5 h (B) were measured. Untreated: tardigrades without chemical treatment; dimethylsulfoxide; those treated with 1% dimethylsulfoxide for 24 h; water: those treated with MQ water for 5 h. *Statistical significance between samples (Tukey–Kramer test; *P* < 0.05). Data represent the mean ± SD; *n* = 3.

Because D942 and AICAR showed different effects on desiccation tolerance of *H. exemplaris*, we next investigated the effects of those chemicals on AMPK kinase activity. In tardigrades treated with D942 for 24 h, no significant change of AMPK kinase activity was observed compared to the untreated control (Fig. 2A). By contrast, treatment with AICAR for 5 h significantly upregulated AMPK kinase activity (Fig. 2B). Moreover, because the primary binding protein of D942 was NQO1 of mitochondrial complex I in the L6 cell lysate and mitochondrial fraction [5], we investigated the presence of this gene in the *H. exemplaris* genome. A similarity search of human NQO1 using blastp revealed that there appeared to be no NQO1 in *H. exemplaris* (threshold *e*‐value: 0.1), confirming previous reports that this gene is not conserved in invertebrates [11]. These results suggest that improved desiccation tolerance by treatment with D942 might be a result of currently unknown mechanisms but not the activation of AMPK kinase activity.

### Proteome of tardigrades treated with D942

**Fig. 3:**
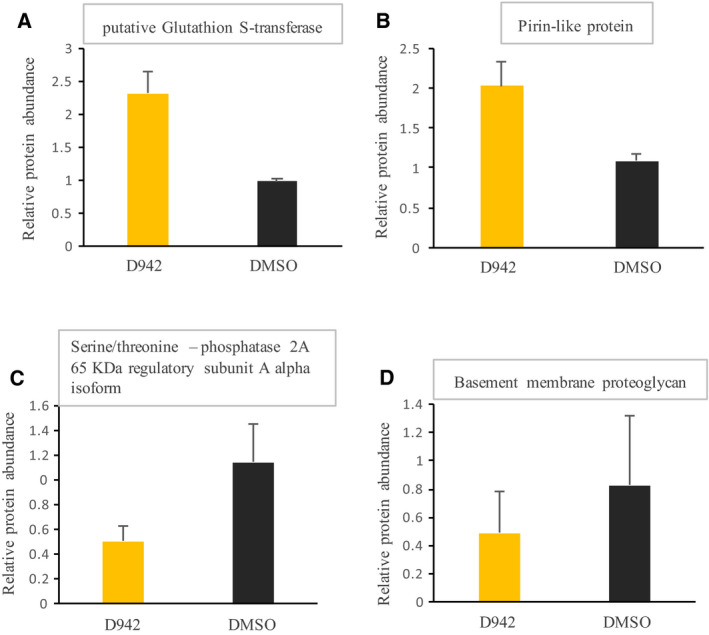
Significant proteins possibly involved in improving desiccation tolerance. Proteins for which amounts were upregulated (A, B) or downregulated (C, D) by D942 treatment. Protein levels were normalized with respect to untreated (without chemical treatment). Error bars represent the SD. Additional details are provided in the Materials and methods.

Because tardigrades needed at least more than 5 h of D942 treatment to achieve increased desiccation tolerance (Fig. 1C), we assumed that *de novo* protein synthesis might be involved. Therefore, to elucidate the underlying mechanisms of improved desiccation tolerance by D942 treatment, we performed proteomics of tardigrades treated with D942 for 24 h. In total, 1529 proteins were detected and, of those, 148 proteins were differentially regulated proteins (Table S1). To improve data reliability, we selected the significant proteins from differentially regulated proteins using strict criteria (see Materials and methods) and identified four proteins for which protein abundance were reliably changed by D942 treatment. Putative glutathione *S*‐transferase and pirin‐like protein were upregulated and serine/threonine‐protein phosphatase 2A (PP2A) 65‐kDa regulatory subunit A alpha isoform and basement membrane proteoglycan were downregulated (Fig. 3 and Table S1). Because both upregulated proteins were somewhat related to cellular oxidative states, we attempted to measure GSH‐to‐GSSG ratios in tardigrades that were untreated, dimethylsulfoxide‐treated and D942‐treated and then aimed to compare these with each another. Concentrations for total glutathione, mainly consisting of GSH, were obtained, whereas those for GSSG were not as a result of concentrations of GSSG being too small, although 2000 tardigrades contributed to each of them (Fig. S3).

## Discussion

In a previous study, we showed that pre‐treatment with the AMPK inhibitor (compound C) significantly inhibited anhydrobiotic survivals in *H. exemplaris* and thus AMPK activity is required for successful anhydrobiosis in this tardigrade species [4]. Accordingly, we assumed that upregulation of AMPK activity would contrarily increase the desiccation tolerance of *H. exemplaris*. In the present study, we therefore investigated the effects of two known AMPK activators for which the mechanisms of actions are distinct. Intriguingly, tardigrades pre‐treated with AMPK indirect activator (mammalian mitochondrial complex I inhibitor; D942) increased desiccation tolerance (Fig. 1A–C), whereas AMPK direct activator (AICAR) scarcely affected desiccation tolerance (Fig. 1D and Fig. S1). The difference in solvents, dimethylsulfoxide or MQ water is likely not the cause for these distinct outcomes because tardigrades soaked in either solution showed similar recovery rates (Fig. 1 and Fig. S1). It is plausible that no significant activation of AMPK kinase activity by D942 treatment was observed (Fig. 2A) because *nqo1* was not detected in *H. exemplaris* genome by a blastp search. In the previous study, which shows that NQO1 was one of the binding proteins of D942 in the L6 cell lysate and mitochondrial fraction, eight other primary binding proteins were identified, although the binding specificities of those remain to be investigated [5]. Those remaining eight proteins could be candidates for binding protein in tardigrades. Although the binding protein(s) of D942 in tardigrades remain(s) unknown, proteomics of tardigrades treated with D942 identified some proteins possibly involved in enhanced desiccation tolerance. In the proteomics, out of 1529 detected proteins, there were only four proteins for which expression levels were changed under strict criteria (Fig. 3 and Table S1), suggesting that only a few proteins might be involved. The two upregulated proteins, putative glutathione *S*‐transferase and pirin‐like protein, are known to be associated with the response to oxidative stress. Glutathione S‐transferase is involved in the detoxification of not only exogenous but also endogenous molecules including oxidized lipids [12]. The mRNA levels of glutathione S‐transferase were also increased by desiccation stress [3], suggesting that this gene plays an important role in desiccation tolerance in *H. exemplaris*. Pirin‐like protein is indicated to modify the metabolism of long fatty acid chain in redox‐sensitive manner [13]. Taken together, both proteins might play roles with respect to reducing the cellular oxidative damage caused by desiccation stress. This could be supported by the well‐documented notion of the association between oxidative damage and desiccation tolerance [14, 15, 16, 17]. For example, Pereira *et al*. [17] demonstrated that yeast cells were oxidized more than 10‐fold by desiccation. Desiccated organisms kept in a vacuum or a reduced oxygen environment such as nitrogen recovered well compared to those kept in normal air or oxygen environment, suggesting that exogenous oxidation is a damaging factor during anhydrobiosis [18, 19, 20]. On the other hand, there were two downregulated proteins. One of them is PP2A 65‐kDa regulatory subunit A alpha isoform. In the previous study, the activity of PP2A was suggested to be required for successful anhydrobiosis in *H. exemplaris* [2]. PP2A is a tetramer consisting of a scaffold, as well as catalytic and regulatory subunits [21, 22]. Various regulatory subunits determine the specificity to various biological processes, and therefore downregulation of this regulatory subunit might modify the activity of PP2A, which is associated with desiccation stress tolerance. Proteoglycan is a main component of the extracellular matrix [23]. In *H. exemplaris* exposed to mild desiccation stress, the abundance of this protein was also significantly reduced [4]. It is intriguing that D942 treatment induced the same change as mild desiccation treatment on the amount of proteoglycan. Considering that *H. exemplaris* requires tun formation for successful anhydrobiosis, downregulation of basement membrane proteoglycan might contribute to facilitating transformation of dehydrating tardigrades into tun. The association between D942 treatment and tun formation will be another direction for future exploration.

We also investigated whether D942 treatment was able to confer desiccation tolerance to a desiccation‐intolerant tardigrade from the same family Hypsibiidae, *T. ruffoi* species, which is exclusively aquatic [24]. The common ancestor of the order Parachela likely possessed the ability to go into anhydrobiosis because anhydrobiosis is conserved across the two orders within the class Eutardigrada, and *T. ruffoi* has probably undergone a secondary loss of this mechanism [16]. Because treatment with D942 conferred desiccation tolerance to *H. exemplaris* bypassing preconditioning, we investigated the possibility of a similar gain of tolerance in this intolerant species. As shown in Fig. S2, *T. ruffoi* species, as maintained in our laboratory, was intolerant against exposure at even 97% RH, indicating that this species is desiccation‐intolerant. We revealed that D942 treatment had no effect on their desiccation tolerance (Fig. S2). This observation suggests that D942 treatment is not sufficient to achieve desiccation tolerance in desiccation‐intolerant organisms.

D942 is the first chemical to be identified that can increase the desiccation tolerance of an anhydrobiotic tardigrade. Further studies aiming to elucidate the underlying mechanisms, especially focusing on oxidative stress during dehydration, would enhance our understanding of the molecular mechanisms of desiccation tolerance not only in tardigrades, but also in other organisms.

## Conflict of interests

The authors declare that they have no conflicts of interest. The funders had no role in the design of the study; in the collection, analyses, or interpretation of data; in the writing of the manuscript; or in the decision to publish the results.

## Author contributions

KK and KA conceived and designed the project. KK and MM acquired the data. KK and KA analyzed and interpreted the data. KK, MT and KA acquired funding. KK and KA wrote original draft. KK, MM, MT and KA reviewed and edited the manuscript submitted for publication.

## Supporting information


**Fig. S1.** Effects of AICAR on desiccation tolerance.
**Fig. S2.** Effects of D942 on desiccation tolerance in *Thulinius ruffoi*.
**Fig. S3.** Quantification of glutathione (GSH‐to‐GSSG).

**Table S1.** Proteome of tardigrades treated with D942.

## Data Availability

All of the data presented in this manuscript are available from the corresponding author upon reasonable request.
