# Supplementary material for: Pre‐treatment with D942, a furancarboxylic acid derivative, increases desiccation tolerance in an anhydrobiotic tardigrade Hypsibius exemplaris
Source: FEBS Open Bio. 2020 Jul 22;10(9):1774–81. doi: 10.1002/2211-5463.12926 (PMC7459401; doi:10.1002/2211-5463.12926)
Supplement: Supplementary file 1 — Fig. S1. Effects of AICAR on desiccation tolerance. Fig. S2. Effects of D942 on desiccation tolerance in Thulinius ruffoi. Fig. S3. Quantification of glutathione (GSH‐to‐GSSG). [file FEB4-10-1774-s001.pdf]

(a)

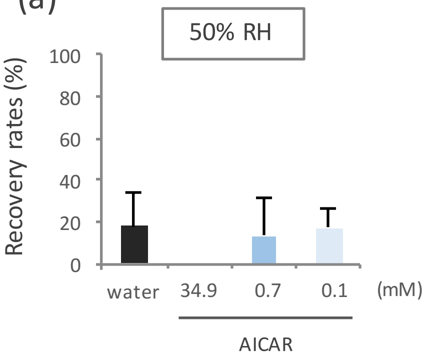

Fig S1. Effects of AICAR on desiccation tolerance

(a) Recovery rates of tardigrades, *H. exemplaris* pre-treated with AICAR at different concentrations or MQ water for 24 h and subsequently desiccated at 50% RH for 2 days. Data represents mean ± SD; N=3; 15 animals each.

(a)

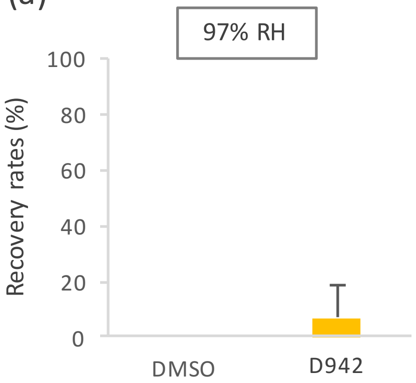

Fig S2. Effects of D942 on desiccation tolerance in *Thulinius ruffoi*

(a) Recovery rates of tardigrades pre-treated with D942 (1 mM) or 1% DMSO for 24 h and subsequently desiccated at 97% RH for 2 days. Data represents mean ± SD; N=3; 15 animals each.

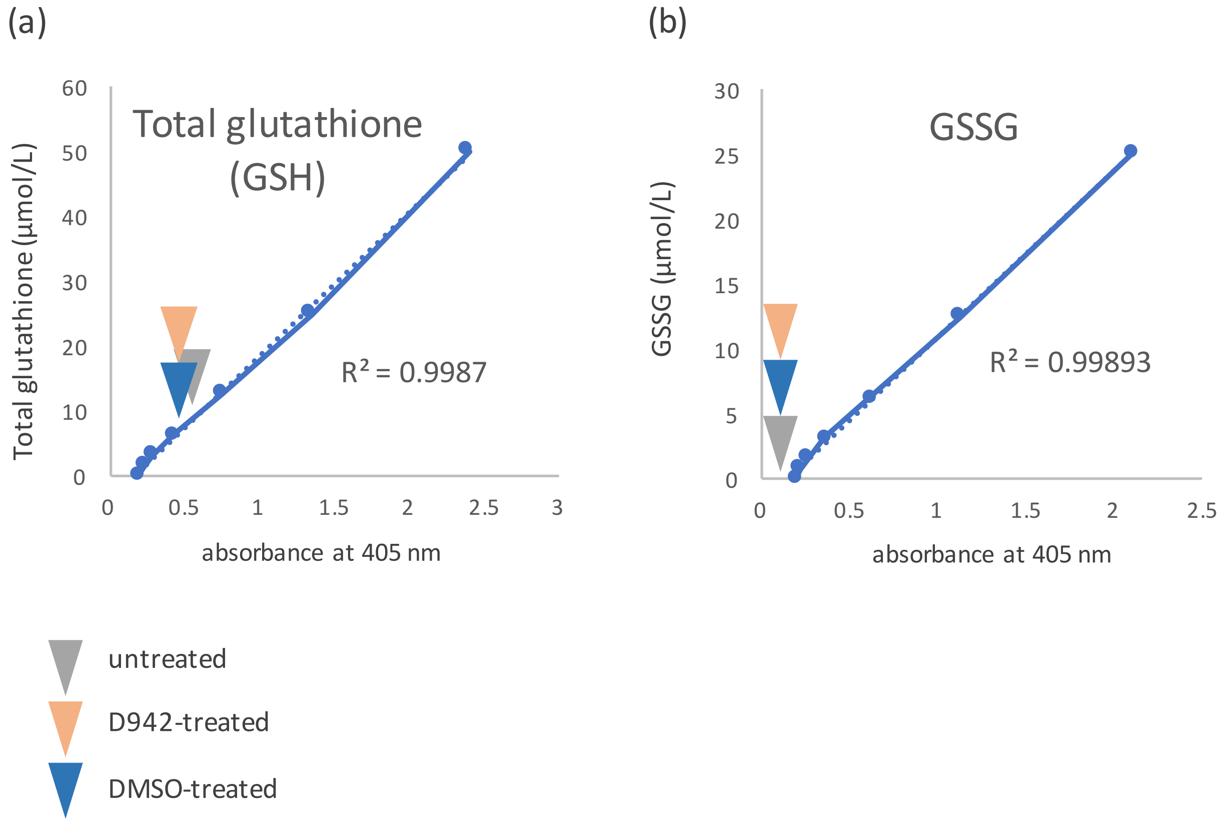

Fig S3. Quantification of Glutathione (GSH-to-GSSG)

A linear trend line was generated by standards of GSH (a) and of GSSG (b). The range of standards for quantification was 0.5 - 50 µmol/L total glutathione (GSH) and 0.5 - 25 µmol/L GSSG. R^2 represents correlation coefficients. Two thousands tardigrades (*H. exemplaris*) were collected without any chemical treatment (untreated, gray arrow) or after soaking in 1mM D942 (orange arrow) or 1% DMSO (blue arrow) solutions for 24 h (N=1) and subjected to quantification. GSSG in any group was out of quantification.
